# Mirror Aneurysms of the Pericallosal Artery Clipped During a Single Surgical Procedure: Case Report and Literature Review

**DOI:** 10.3390/jcm13226719

**Published:** 2024-11-08

**Authors:** Corneliu Toader, Mugurel Petrinel Radoi, Felix-Mircea Brehar, Matei Serban, Luca-Andrei Glavan, Razvan-Adrian Covache-Busuioc, Alexandru Vlad Ciurea, Nicolae Dobrin

**Affiliations:** 1 Department of Neurosurgery “Carol Davila”, University of Medicine and Pharmacy, 050474 Bucharest, Romania; corneliu.toader@umfcd.ro (C.T.); matei.serban2021@stud.umfcd.ro (M.S.); luca-andrei.glavan0720@stud.umfcd.ro (L.-A.G.); razvan-adrian.covache-busuioc0720@stud.umfcd.ro (R.-A.C.-B.); prof.avciurea@gmail.com (A.V.C.); 2 Department of Vascular Neurosurgery, National Institute of Neurology and Neurovascular Diseases, 077160 Bucharest, Romania; 3 Department of Neurosurgery, Clinical Emergency Hospital “Bagdasar-Arseni”, 041915 Bucharest, Romania; 4 Neurosurgery Department, Sanador Clinical Hospital, 010991 Bucharest, Romania; 5 “Nicolae Oblu” Clinical Hospital, 700309 Iasi, Romania; dobrin_nicolaie@yahoo.com

**Keywords:** pericallosal artery aneurysm, mirror aneurysms, multiple aneurysms, microsurgery, aneurysm microclipping

## Abstract

Pericallosal artery aneurysms are rare, accounting for 2–9% of all intracranial aneurysms, and mirror aneurysms in this location are exceptionally uncommon, presenting unique surgical challenges due to their deep location and proximity to critical neurovascular structures. The aim of this case report is to describe the surgical management and successful outcome of a patient with mirror pericallosal artery aneurysms and to contribute insights into the clinical and surgical considerations for this rare condition. We report the case of a 71-year-old female with multiple cardiovascular and metabolic conditions, including hypertension and smoking—well-established risk factors for intracranial aneurysm formation and rupture. She presented with a Hunt and Hess grade II subarachnoid hemorrhage resulting in communicating internal hydrocephalus. Preoperative angiography revealed mirror aneurysms of the pericallosal artery. The patient underwent a left basal paramedian frontal craniotomy, during which a ruptured aneurysm on the right A2 segment and an unruptured aneurysm on the left A2 segment were identified. Both aneurysms were successfully clipped using curved Yasargil clips. Postoperative recovery was favorable, with no neurological deficits and stable imaging findings at a three-month follow-up. This case underscores the necessity for precise microsurgical intervention and a thorough understanding of pericallosal artery anatomy to manage such rare and challenging conditions effectively. The role of hyperlipidemia and statin use in intracranial aneurysm development remains debated and warrants further investigation. Our successful management of mirror pericallosal artery aneurysms contributes to the limited literature on this rare condition and highlights the importance of meticulous surgical techniques for favorable outcomes.

## 1. Introduction

Intracranial or cerebral aneurysms, also referred to as cerebral aneurysms, are abnormal swelling of blood vessels in the brain that form when blood flow pressure on weakened arterial or venous walls causes protrusion of brain tissues into space. While intracranial aneurysms can remain asymptomatic for extended periods of time, rupture could pose significant risks that could result in subarachnoid hemorrhage (SAH) with potentially devastating neurological consequences or even cause fatality. SAH occurs when a ruptured aneurysm leads to bleeding in the space between the brain and the surrounding membrane (the subarachnoid space). This can cause severe brain damage and increased intracranial pressure, and if it is untreated, it can be fatal. SAH is one of the most dangerous types of stroke and often results in long-term neurological impairment [1].

Unruptured intracranial aneurysms (UIAs) in the general population have an estimated prevalence rate of 2.8% (95% CI: 2.0–3.9%) with an incidence rate of 15.6 per 100,000 persons [2]. SAH cases were also observed at 7.7 per 100,000 persons within this same population; globally speaking, the incidence rate is estimated at approximately 3.2% [3]. Gender disparity exists when it comes to UIAs and SAH cases, with women more often experiencing them. The female-to-male ratio for aneurysmal subarachnoid hemorrhage aneurysms is nearly 2:1, while overall, brain aneurysms occur 60% more often among women than among males [4,5,6,7].

Age plays a significant role in the prevalence of UIAs and SAH. The highest incidence rate for UIAs occurs among those aged 75–84 years, at 61.6 per 100,000 persons. SAH cases peak among individuals 85 years or older at 30.1 per 100,000 persons; their prevalence increases gradually with each decade of life, culminating in their peak around age 60 [2,8].

Intracranial aneurysms have been linked with several genetic conditions, including autosomal dominant polycystic kidney disease, neurofibromatosis type I, Ehlers–Danlos syndrome, Loeys–Dietz syndrome, and Marfan syndrome [9]. Genetic predisposition plays a significant role in intracranial aneurysms; having a family history increases your risk significantly [10]. Smoking and hypertension independently also increase risk; each increases by an odds ratio of 3.0; when combined, however, their combined effect amplifies risk significantly—an odds ratio of 8.3 for intracranial aneurysms and 15-fold for subarachnoid hemorrhages [11,12].

Female sexual activity after menopause can increase vulnerability to intracranial aneurysms [13]. Risk increases with age, peaking between the sixth and eighth decades [14].

This case report presents a unique instance of mirror pericallosal artery aneurysms, a rare condition that poses significant surgical challenges due to its deep location and the complex vascular anatomy involved. The simultaneous management of ruptured and unruptured aneurysms in the pericallosal artery highlights not only the need for precise microsurgical techniques but also the importance of early diagnosis and intervention. Our report contributes novel insights into the successful management of this rare pathology, adding to the limited literature on pericallosal aneurysms and providing a detailed analysis of the surgical approach and outcome. This case underscores the need for further exploration into the role of vascular risk factors and how they interact with genetic predispositions to influence aneurysm development and rupture risk.

## 2. Case Presentation

A 71-year-old female patient with a history of multiple cardiovascular and metabolic conditions was first admitted to our clinic for the surgical treatment of mirror aneurysms of the pericallosal artery. The patient had presented with a Hunt and Hess II subarachnoid hemorrhage, leading to communicating internal hydrocephalus.

**Figure 1:**
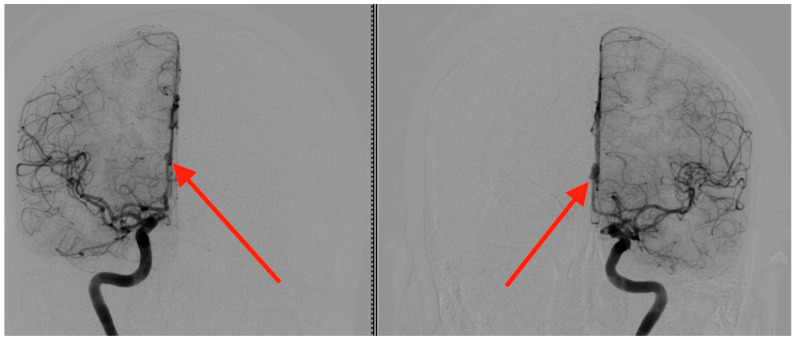
Frontal angiographic view displaying two mirror aneurysms of the pericallosal artery. The left panel demonstrates a ruptured pericallosal artery aneurysm (indicated by the red arrow), with clear evidence of vascular disruption and extravasation patterns consistent with rupture. The right panel shows an unruptured pericallosal artery aneurysm (highlighted by the red arrow), where the vascular structure remains intact, lacking signs of hemorrhage or leakage.

**Figure 2:**
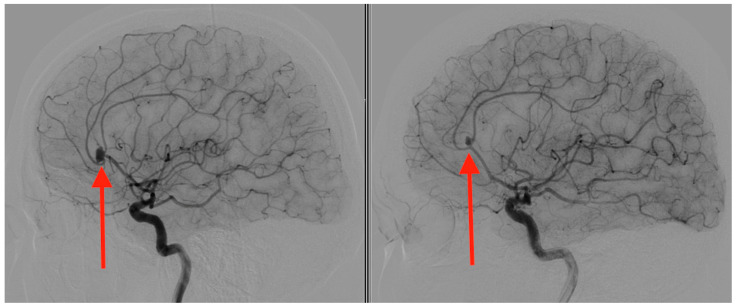
Sagittal section angiography illustrating two mirror aneurysms of the pericallosal artery. The left panel depicts a ruptured pericallosal artery aneurysm (indicated by the red arrow), with evidence of vascular compromise and associated extravasation. The right panel shows an unruptured pericallosal artery aneurysm (highlighted by the red arrow), with a smooth vascular contour and no indications of hemorrhage. Under the operating microscope, an interhemispheric approach was taken for surgical intervention, during which numerous blood clots were observed and evacuated.

**Figure 3:**
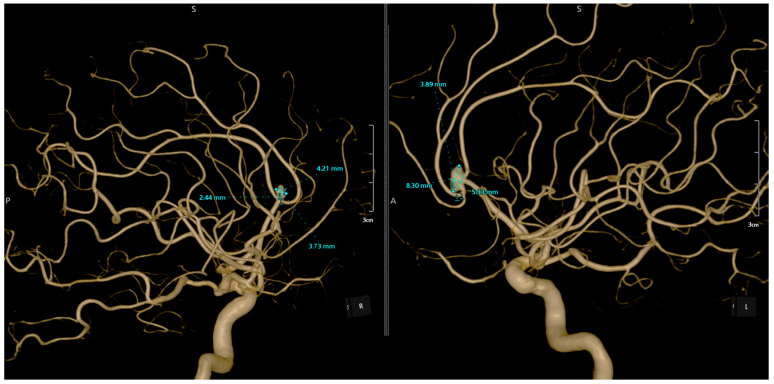
Angiographic reconstruction of the two mirror aneurysms. Angiographic reconstruction showing the mirror aneurysms. The ruptured aneurysm on the right A2 segment is clearly visible, along with the unruptured aneurysm on the left A2 segment, which was surgically clipped. A curved clip (size 6.3) was placed on its neck. Dissection continued, revealing another unruptured aneurysm at the bifurcation of the left A2 segment, located inferior to the ruptured one and below and anterior to the genu of the corpus callosum. This aneurysm, obstructing the view of the bilateral A2 segments, measured approximately 8.5 mm in maximum diameter, and a curved Yasargil clip was applied to its neck. Hemostasis was achieved using electrocoagulation and Surgicel. Moderate cerebral collapse was noted.

Pre-operative angiography highlighted the aneurysms (Figure 1, Figure 2 and Figure 3).

A basal paramedian frontal craniotomy was performed on the left side, with the medial margin at the anterior third of the superior sagittal sinus (SSS). The dura mater, under tension, was extremely thin and adhered to the bone flap, and thus remained closely attached to the bone flap, which was elevated without incidents or venous drainage system interception. The brain was noted to be edematous and intensely reddish, tending to herniate through the dural opening, prompting a ventricular puncture for relaxation.

A dural defect remained, with duroplasty deemed unfeasible. The bone flap was replaced over a drain that was externalized through a burr hole. The scalp was sutured in two layers, and a dressing was applied.

The patient’s medical history was significant for effort-induced angina pectoris (CCS class II), mild cerebral vasospasm, urinary sphincter incontinence, grade I obesity, difficult intubation, spontaneously resolved nasal CSF fistula, grade II essential hypertension with a high additional risk for cardiovascular events such as stroke or myocardial infarction, ischemic heart disease, cerebral abiotrophic syndrome, and cerebral and systemic atherosclerosis.

**Figure 4:**
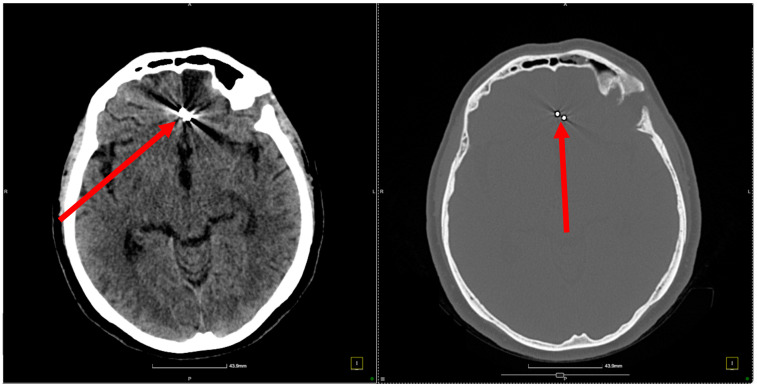
Follow-up control CT scan. In the left panel (soft tissue window), the red arrow indicates the location of the surgical clip within the anterior frontal interhemispheric and pericallosal cisterns. This clip was placed to secure the aneurysm and prevent further rupture. The surrounding radiopaque artifacts are a result of the metallic clip, which creates streaks that partially obscure adjacent soft tissue structures but serve as a clear marker of the clip’s position. In the right panel (bone window), the red arrow points to the same surgical clip, with a clearer view of its orientation and position relative to the skull. This panel illustrates the stability of the clip placement and provides additional confirmation of its location in the anterior frontal region. The bone window setting reduces soft tissue detail but helps assess any potential impact on bony structures, with no evidence of abnormalities in adjacent cranial bones.

**Figure 5:**
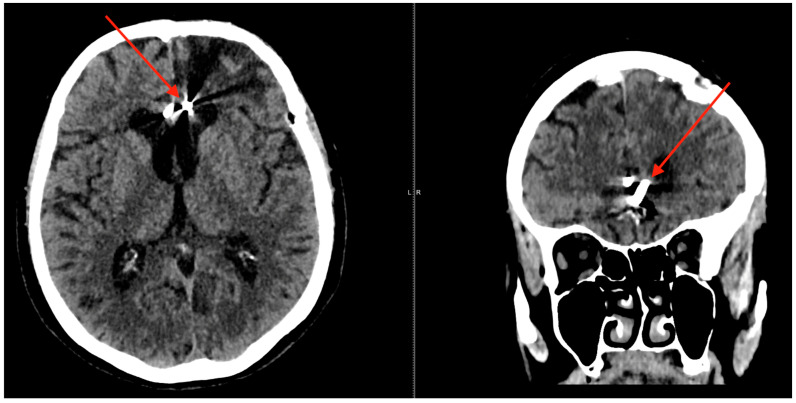
Follow-up control CT scan comparing transversal (**left**) and frontal (**right**) sections, showing the surgical clips within the anterior frontal interhemispheric and pericallosal cisterns. The red arrows mark the clip location in both views, confirming its precise and stable placement in the interhemispheric fissure, providing a clear spatial reference for post-operative evaluation.

**Figure 6:**
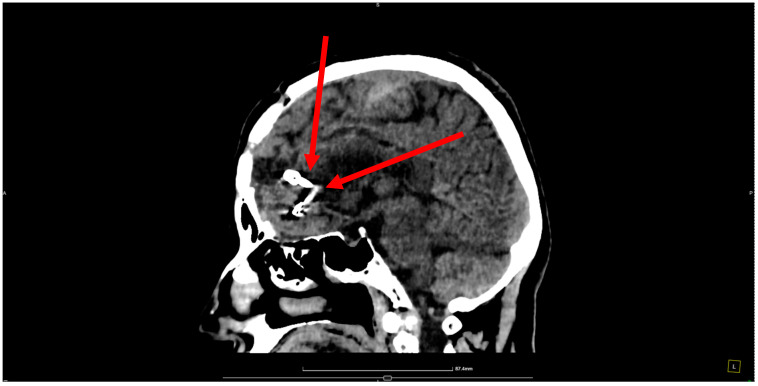
Follow-up control CT scan, sagittal section, illustrating the artifacts from two 2 × 6.3 mm curved Yasargil clips. The red arrows highlight the locations of the clips within the anterior frontal interhemispheric and pericallosal cisterns. The metallic nature of the clips produces radiopaque artifacts, providing clear markers of clip placement and alignment. This image offers a sagittal perspective to assess the spatial orientation and artifact effects of the Yasargil clips in the post-operative region.

**Figure 7:**
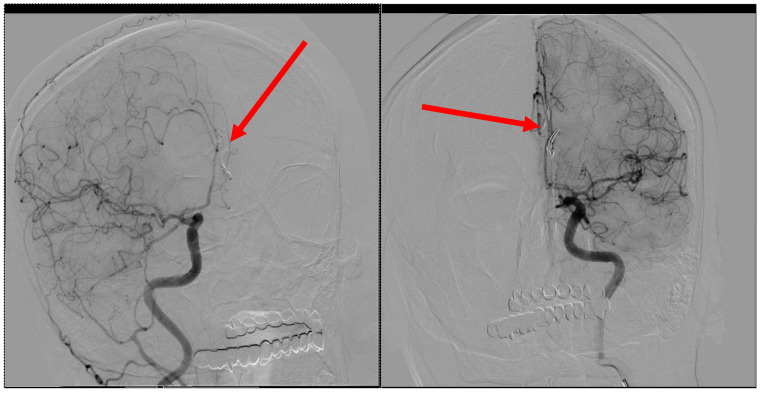
Follow-up contrast angiography illustrating the placement of two microsurgical clips (2 × 6.3 mm curved Yasargil clips). The left panel shows one of the clips, indicated by the red arrow, positioned within the pericallosal artery area. The right panel provides another view of the second clip, also marked by a red arrow, confirming its secure placement. Both panels highlight the precise positioning and orientation of the Yasargil clips, used for aneurysm stabilization. The angiographic images effectively demonstrate the integrity and stability of the clips in the post-operative vascular architecture.

Upon readmission for clinical and imaging reevaluation 3 months later, the patient was conscious, coherent, and cooperative, with no neurological deficits, language disturbances, or sensory impairments. A control CT scan revealed artifacts from the presence of clips in the anterior frontal interhemispheric and pericallosal cisterns, a small hypodense sequelae area in the anterior left frontal region, moderate cerebral atrophy, and a normally sized ventricular system aligned with the midline (Figure 4, Figure 5 and Figure 6). Moreover, control angiography showed the correct placement of the clips (Figure 7). There were no signs of recent hemorrhage.

Overall, the patient’s postoperative course was favorable, with significant neurological improvement, and the current evaluation showed stable postoperative changes without new complications.

## 3. Discussion

Approximately 85% of saccular aneurysms occur within the circle of Willis. Anterior communicating artery aneurysms, most frequently located within the circle of Willis, account for about 35% of intracranial aneurysms; aneurysms related to the carotid arteries—including the internal carotid artery, posterior communicating artery, and ophthalmic artery—as well as those involving the internal carotid artery, account for about 30% of all intracranial aneurysms. Middle cerebral artery aneurysms represent 22%. Finally, posterior circulation aneurysms, typically found near the basilar artery tips, may still have significant clinical implications despite being less frequent [15].

Pericallosal artery aneurysms represent 2–9% of all intracranial aneurysms. Most frequently, they form at the bifurcation of the pericallosal and callosomarginal arteries; less frequently, they can also form further along its course, such as more distal segments of the pericallosal artery. They exhibit a higher rupture rate compared with other anterior circulation intracranial aneurysms and often present with pericallosal intracerebral hematomas, indicative of their propensity for severe clinical manifestations. Furthermore, surgery management of pericallosal aneurysms often brings with it high complication rates, further emphasizing their significant difficulty [16,17].

Lee and colleagues highlighted that effective surgical treatment of Pericallosal artery (PA) aneurysms requires a deep knowledge of their microsurgical anatomy, avoiding potential pitfalls, and drawing upon experience to successfully manage them [18]. Therefore, we took the necessary steps for safe and successful clip placement, which included performing an atraumatic opening of the interhemispheric fissure, protecting major draining veins, accurately localizing aneurysms, and early exposure of proximal segment A2 of the anterior cerebral artery.

Bilateral PA aneurysms in mirror positions represent an extremely rare clinical scenario; Niijima and colleagues reported one of these rare cases—that of a 47-year-old female suffering from ruptured bilateral PA aneurysms located on both sides, which were successfully treated [19]. Another case was represented by a 46-year-old female with a history of smoking 20 cigarettes per day. Her mirror aneurysms were treated using microsurgical clipping [19].

In our case, our 71-year-old patient also had a history of smoking and uncontrolled hypertension, both of them being key risk factors in the development of intracranial aneurysms. Moreover, females are more prone to developing intracranial aneurysms [20,21]. Another interesting note is the fact that our patient was known to have had a myocardial infarct, for which she was taking statins. The link between hyperlipidemia, statin use, and intracranial aneurysms is debated. Lipid accumulation, particularly of Low-Density Lipoprotein (LDL), in aneurysmal walls has been implicated in their degeneration and inflammation processes, two critical elements in aneurysm formation and pathology [22]. Statins, commonly prescribed to lower LDL, possess additional biological effects that may help strengthen aneurysm walls and decrease rupture risk [23]. Zhang et al.’s findings that genetic polymorphisms associated with elevated High-Density Lipoprotein (HDL) levels are linked to reduced risks of intracranial aneurysm formation and rupture suggest lifestyle modifications aimed at increasing HDL may reduce subarachnoid hemorrhage risk, and similarly, the correlation between elevated LDL levels and IA formation is notable [24]. To this day, no clear answer has been given on the topic of lipids and intracranial aneurysms, and it is clear that more research needs to be conducted in this regard [25].

A comprehensive literature search was conducted using databases including PubMed, Google Scholar, and ScienceDirect. Search terms such as ‘pericallosal artery aneurysm’, ‘mirror aneurysms’, ‘intracranial aneurysm microsurgery’, and ‘hyperlipidemia and aneurysm risk’ were used. The search was limited to articles published between 1980 and 2023.

Recent studies (Table 1) indicate that while microsurgical clipping remains a viable option for managing mirror aneurysms, endovascular approaches are increasingly being used, especially for aneurysms located in deep cerebral structures [25,26,27]. While the risk factors for intracranial aneurysms, such as smoking and hypertension, are well established, emerging research highlights the potential role of dyslipidemia and genetic predispositions in aneurysm formation and rupture [21,22]. This synthesis of data suggests that patient-specific factors, such as vascular health and genetic background, should be considered when determining the optimal surgical approach.

The table provides a comprehensive review of studies focusing on mirror aneurysms of the pericallosal artery that were surgically clipped during a single procedure. It includes data from six studies published between 1997 and 2022, covering key details such as the number of patients, sex ratio, aneurysm localization, dimensions, neck size, feeding arteries, types of clips used, and follow-up periods.

The number of patients varies across studies, ranging from 14 to 47, reflecting the variability in case complexity and availability of cases. The sex ratio shows a balanced distribution in some studies, while others report a higher incidence in males, although there is no consistent pattern indicating a strong gender predisposition.

Aneurysm localization is primarily focused on the distal anterior cerebral artery (ACA), particularly in areas such as the A2–A3 junction, A3 segment, and pericallosal artery bifurcation, where mirror aneurysms affect both sides of the ACA. The aneurysm dimensions range from 3 mm to 10 mm, with larger aneurysms generally presenting a higher surgical challenge.

Neck dimensions, ranging from 2 mm to 6 mm, play a crucial role in determining the surgical approach and clip selection. Wider-necked aneurysms often require specialized clips, such as fenestrated or tandem clips. The feeding arteries are typically the pericallosal artery or its branches, such as the callosomarginal artery, with some cases involving bifurcations at the A2–A3 junction.

Various types of clips were used across the studies, including standard titanium clips, fenestrated clips, and tandem clips, depending on the complexity of the aneurysm’s anatomical structure. The follow-up periods range from 6 to 24 months, with most studies employing imaging techniques like MRI and angiography to assess aneurysm obliteration and monitor for complications.

This table highlights the diversity of surgical techniques and outcomes in treating mirror aneurysms of the pericallosal artery, emphasizing the challenges posed by their location and complex vascular anatomy.

## 4. Conclusions

Our manuscript illustrates the case of a 71-year-old female with mirror pericallosal aneurysms treated using microsurgical clipping. This rare and challenging condition required precise surgical techniques, including careful microsurgical navigation and clip placement.

Key risk factors such as hypertension and smoking were present in the patient, aligning with well-established contributors to the formation and rupture of intracranial aneurysms. While hyperlipidemia and statin use remain debated topics regarding their role in aneurysm formation, they present an intriguing aspect of risk factor evaluation.

Further research is necessary to clarify their impact on the development and complications of intracranial aneurysms. The patient’s postoperative course was favorable, with no neurological deficits and stable imaging findings. This case adds to the limited literature on mirror aneurysms of the pericallosal artery, emphasizing the need for ongoing exploration of both surgical techniques and the broader understanding of associated risk factors.

## Figures and Tables

**Table 1:** This table summarizes the surgical treatment of mirror aneurysms of the pericallosal artery, including aneurysm size, neck dimensions, clip type, feeding artery, and follow-up outcomes in various clinical cases. It provides an overview of surgical approaches and postoperative results.

Study (Author, Year)	Number of Patients	Sex Ratio (M:F)	Aneurysm Localization	Aneurysm Dimensions	Neck Dimensions	Feeding Artery	Type of Clip Used	Follow-Up
Tang et al., 2022 [26]	32	19:13	Distal ACA, including mirror aneurysms	Mean: 5.5 mm	Range: 2.8–4.3 mm	Callosomarginal and pericallosal	Titanium with fenestrations	6–18 months (CT and MRI)
Deuschl et al., 2020 [27]	22	13:9	Pericallosal artery bifurcation	3–8 mm	3–5 mm	Callosomarginal artery	Yasargil clips	24 months (clinical and imaging)
Lehecka et al., 2008 [28]	47	27:20	Distal ACA, mirror aneurysms at A2–A3 junction	Range: 4–10 mm	3–6 mm	A2 segment	Tandem clips (for complex anatomy)	12–24 months (angiography, MRI)
Dashti et al., 2007 [29]	30	17:13	Distal ACA, mirror aneurysms	4–8 mm	2–4 mm	A3 segment	Tandem clips	12 months (angiography)
Inci et al., 1998 [30]	14	9:5	Distal anterior cerebral artery (A3 segment)	Range: 3–8 mm	2–4 mm	Distal ACA (A3)	Standard titanium clips	12 months (MRI and angiography)
Proust et al., 1997 [31]	43	25:18	Pericallosal artery aneurysms, bifurcation	Average 6.2 mm	Mean: 3.5 mm	Pericallosal artery trifurcation	Yasargil clips (wide-necked)	24 months (clinical and imaging)

ACA: Anterior cerebral artery; MRI: Magnetic Resonance Imaging.

## Data Availability

Data are available upon reasonable request from the corresponding authors.
